# Postoperative groin wound complications after common femoral artery exposure are associated with increased long-term systemic and limb adverse outcomes in a national propensity-matched analysis

**DOI:** 10.1016/j.jvscit.2026.102285

**Published:** 2026-04-30

**Authors:** Koyal Ansingkar, Robert Burns, Mauricio Osuna, Daanish Sheikh, Amirtha Shekar, Maham Rahimi

**Affiliations:** a School of Engineering Medicine, Texas A&M University, Houston, TX; b Long School of Medicine, University of Texas Health Science Center at San Antonio, San Antonio, TX; c DeBakey Heart and Vascular Center, Houston Methodist Hospital, Houston, TX

**Keywords:** Postoperative groin complications, Groin incision, Common femoral artery exposure, Long-term outcomes

## Abstract

**Objectives:**

Common femoral artery (CFA) exposure is a critical step in many vascular procedures. Although local postoperative complications are well described, their association with long-term high-acuity sequelae remains incompletely characterized. This study aimed to evaluate the relationship between hematoma, seroma, surgical site infection, and wound necrosis/dehiscence, and the subsequent development of acute respiratory failure, acute kidney injury (AKI), stroke (cerebrovascular accident), ST-elevation myocardial infarction, limb loss, and mortality.

**Methods:**

A retrospective cohort study was conducted using the TriNetX database, identifying patients who underwent CFA exposure. Patients were stratified based on occurrence of hematoma, seroma, surgical site infection, or necrosis/dehiscence within 6 months, and whether they underwent surgical correction of the complication within 3 months. The 1- and 5-year incidences of high-acuity sequelae were analyzed. Propensity score matching controlled for demographics and comorbidities, including hypertension, diabetes, ischemic heart disease, end-stage renal disease, and tobacco use. Relative risk ratios (RRs) with 95% confidence intervals were calculated.

**Results:**

Among 129,774 patients, wound necrosis/dehiscence was the most prevalent complication and strongest predictor of long-term adverse outcomes. Complication rates generally peaked within the first postoperative month. Any postoperative complication significantly increased the 1-year incidence of limb loss (RR, 4.43; *P* < .0001) and AKI (RR, 1.42; *P* < .0001), as well as the 5-year incidence of limb loss (RR, 3.61; *P* < .0001), acute respiratory failure (RR, 1.28; *P* < .0001), AKI (RR, 1.28; *P* < .0001), cerebrovascular accident (RR, 1.09; *P* = .027), and mortality (RR, 1.03; *P* = .044). Patients requiring corrective procedures experienced additional risk, including higher 1-year and 5-year rates of limb loss and AKI.

**Conclusions:**

Postoperative groin wound complications after CFA exposure are independently associated with increased long-term systemic and limb adverse outcomes, particularly limb loss and AKI. Wound necrosis/dehiscence demonstrated the strongest association across outcomes. These findings suggest that groin complications represent clinically meaningful markers of downstream risk and identify a critical early postoperative window for intensified surveillance and preventive intervention.

**Article Highlights**

• **Type of Research:** Retrospective multicenter propensity score-matched cohort study using the TriNetX national electronic health record database.
• **Key Findings:** Postoperative hematoma, seroma, surgical site infection, and wound necrosis/dehiscence after common femoral artery exposure were associated with increased long-term risks of acute kidney injury, acute respiratory failure, cerebrovascular accident, limb loss, and mortality. Wound necrosis/dehiscence demonstrated the strongest association with adverse outcomes, and patients requiring reintervention had particularly elevated risks of acute kidney injury and limb loss.
• **Take Home Message:** Groin wound complications following open common femoral artery exposure may serve as early indicators of elevated systemic and limb-related risk. Closer surveillance during the early postoperative days may facilitate earlier identification of high-risk patients and improve downstream outcomes.

Open surgical exposure of the common femoral artery (CFA) through a groin incision remains fundamental to open and hybrid vascular procedures, but is associated with a high burden of wound morbidity and downstream systemic risk. In procedures requiring open femoral artery exposure, including lower extremity revascularization and endovascular abdominal aortic aneurysm repair, groin surgical site complication rates approach 20% to 30%, most commonly due to hematoma, seroma, infection, and wound breakdown, many of which require reintervention.^1^, ^2^, ^3^ In a population with substantial baseline cardiovascular and renal vulnerability, these complications may extend beyond local morbidity and contribute to adverse systemic outcomes.^4^, ^5^, ^6^

Hematoma and seroma are among the most frequent early complications after CFA exposure and are reported in up to one-fifth of patients; both strongly predispose to subsequent groin surgical site infections (SSIs).^2^^,^^3^^,^^7^ Groin SSIs are particularly consequential due to prosthetic graft use, reoperative fields, and compromised soft tissue perfusion, and are associated with prolonged hospitalization, increased costs, and higher rates of early reoperation.^1^^,^^3^^,^^7^, ^8^, ^9^ Severe cases may require serial debridement, muscle flap coverage, or graft excision and are linked to limb loss and mortality when prosthetic material is involved.^1^^,^^7^^,^^8^

Groin wound complications are a major driver of early reintervention after femoral exposure.^5^^,^^10^ Nearly one-half of patients who develop a groin SSI undergo reoperation within 30 days, compared with a minority of those without infection.^3^ Larger series demonstrate that hematoma evacuation, wound debridement, revision of arterial reconstruction, redo groin exploration, and soft tissue flap coverage contribute substantially to reintervention rates.^9^ The cumulative burden of repeat procedures, blood loss, and inflammatory stress may represent a mechanistic link between localized groin complications and subsequent systemic events.

Patients undergoing vascular surgery are already at elevated risk for postoperative acute kidney injury (AKI), respiratory failure, neurological events, and cardiovascular complications.^4^^,^^6^ Complicated wound courses characterized by infection, bleeding, and hemodynamic instability may exacerbate this risk and promote multiorgan dysfunction. In peripheral vascular surgery, wound infection and soft tissue failure are associated with major amputation and death, reflecting the combined effects of local sepsis, impaired limb perfusion, and systemic cardiovascular disease.^7^^,^^8^^,^^11^

Using TriNetX, a federated national research network of deidentified electronic health records (EHRs) from diverse health care settings across the United States, we evaluated these associations with sufficient power to detect clinically meaningful increases in postoperative sequelae. Existing evidence suggests that preventive measures, including meticulous hemostasis, optimized wound management, and targeted infection prevention strategies, may reduce groin wound complications; however, the downstream systemic impact of these complications remains incompletely defined.^11^ This study aimed to characterize the relationship between specific groin wound complications and high-acuity downstream outcomes. Understanding the extent to which groin complications serve as markers or mediators of long-term systemic risk is essential to inform perioperative decision-making and postoperative surveillance in contemporary vascular practice.

## Methods

### Data source and study design

TriNetX is a federated research network aggregating deidentified EHR data from health care organizations across the United States, including academic medical centers, community hospitals, and outpatient practices. At the time of this study, the network included 135,735,211 patients from 112 participating health care organizations. The database contains information on demographics, diagnoses (*International Classification of Diseases* codes), procedures (Current Procedural Terminology and Systematized Nomenclature of Medicine – Clinical Terms codes), laboratory results, and medication exposures. Data are maintained through ongoing integration with institutional EHR systems, with quality checks for completeness and consistency. Because TriNetX aggregates real-world clinical data, the population represents a broad spectrum of patients receiving care across diverse health care settings, rather than a claims-only or trial-specific cohort. We conducted a retrospective cohort study using this database to evaluate outcomes after CFA exposure.

### Cohort identification

Patients who underwent open CFA exposure from 2005 to 2025 were identified using standardized *International Classification of Diseases*, 10th edition, Current Procedural Terminology, and Systematized Nomenclature of Medicine – Clinical Terms codes corresponding with open femoral artery exposure or cutdown. CFA exposure was defined as open surgical groin incision with direct arterial dissection and control. Percutaneous femoral access procedures without open arterial exposure were not included. Eligible patients were stratified according to the occurrence of a postoperative wound complication (hematoma, seroma, SSI, or wound necrosis/dehiscence) within 6 months of the index procedure. Pediatric patients were excluded. The codes used to identify CFA exposure are detailed in Supplementary Table I (online only). The codes used to identify postoperative wound complications and codes identifying operative interventions performed to address these complications are provided in Supplementary Table II (online only). Although wound necrosis/dehiscence may occur in the setting of infection and are sometimes classified clinically within the spectrum of SSI, wound necrosis/dehiscence was analyzed separately from SSI in this study. This approach was chosen to account for differences in clinical severity and management, because necrosis or dehiscence often reflect structural wound failure, requiring procedural intervention, whereas SSIs may encompass a broader range of presentations, including superficial inflammatory changes or drainage without tissue breakdown. Codes identifying procedures for the operative correction of postoperative complications for analysis on patients undergoing redo groin incision are identified in Supplementary Table III (online only).

### Index event and outcome definitions

For each analysis, the index event, outcome definitions, and follow-up windows were prespecified. The index event was defined as the CFA exposure procedure. Depending on the specific analysis, the presence or absence of a postoperative wound complication was incorporated into the index exposure definition. Outcome time frames varied by analysis and were selected based on clinical relevance. Patients with a documented outcome of interest before the index event were excluded to limit misattribution and better isolate outcomes temporally associated with the index procedure and/or postoperative wound complication.

### Propensity score matching

To mitigate confounding, propensity score matching was performed in a 1:1 fashion for all analyses. The same covariates were used across all matched comparisons to maintain consistency between the cohorts. Variables included in the propensity score model are detailed in Table I. Pre-existing peripheral artery disease and other vascular-specific comorbidities were not included as variables for propensity score matching, to capture the largest possible sample size and obtain the broadest understanding of groin wound complication prevalence. Matching was conducted within the TriNetX platform using its built-in analytic tools.

**Table I:** Characteristics included in propensity score matching

Category	Characteristic
Age	Age at index
Sex	Male
	Female
	Unknown sex
Race	White
	Black or African American
	Unknown race
	Asian
	Other race
	Native Hawaiian or other Pacific Islander
	American Indian or Alaska Native
Ethnicity	Not Hispanic or Latino
	Unknown ethnicity
	Hispanic or Latino
Diagnoses	Hypertensive diseases
	Ischemic heart diseases
	Type 2 diabetes mellitus
	End-stage renal disease
Social factors	Tobacco use

Each characteristic within each category is listed from largest proportion to smallest.

### Statistical analysis

The TriNetX Compare Outcomes analytic module was used to assess outcome incidence, survival, and measures of association. Risk ratios (RRs) with corresponding 95% confidence intervals (CIs) were calculated for each outcome. Statistical significance was assessed using a two-sided alpha level of 0.05.

### Measuring prevalence and incidence from survival probability

Kaplan-Meier survival probability estimates were obtained from the TriNetX research network, where *S(t)* represents the proportion of patients who had not experienced the outcome of interest by day *t* after the index procedure. The prevalence at each time point was estimated as the proportion of the cohort that had ever experienced the outcome by day *t*. Because TriNetX records first occurrences within a defined follow-up window, this is numerically equivalent to the cumulative incidence and was derived directly from the survival function as:

$$\text{Prevalence}\;\left(t\right)=\text{Cumulative}\;\text{incidence}\;\left(t\right)=1\;-\;S\left(t\right)\text{.}$$

The interval incidence rate was calculated as the number of new events occurring within each day interval divided by the person-time at risk during that interval:

$$\text{IR}\left(t_{1},t_{2}\right)=\Delta CI\;/\;\left(\Delta t\times Ā\right)\text{,}$$

where *ΔCI* is the change in cumulative incidence over the interval *[t*_*1*_*, t*_*2*_*]*, *Δt* is the interval length in days, and *Ā* is the midpoint approximation of the at-risk proportion, estimated as the mean of *S(t*_*1*_*)* and *S(t*_*2*_*)*. Incidence rates are expressed per 10,000 person-days.

Because long-term outcomes were evaluated at fixed intervals after the index CFA exposure, patients were required to survive until the beginning of each outcome window to be included in that analysis. Competing risks were not explicitly modeled, and results should, therefore, be interpreted as associations rather than causal effects.

## Results

### Cohort characteristics

We identified 129,774 adult patients who underwent CFA exposure and met the inclusion criteria. Demographic information for patients in the cohort is provided in Table II.

**Table II:** Demographic characteristics of the cohort of all patients undergoing common femoral artery (CFA) exposure

Characteristics		No. of patients	% of cohort
	Mean age at index, years	64 ± 15.3	
Sex	Male	73,842	60.47
	Female	44,848	36.73
	Unknown sex	3424	2.8
Race	White	81,890	67.06
	Black or African American	18,810	15.41
	Unknown race	13,205	10.81
	Asian	3536	2.9
	Other race	3241	2.65
	Native Hawaiian or other Pacific Islander	943	0.77
	American Indian or Alaska Native	489	0.4
Ethnicity	Not Hispanic or Latino	87,958	72.03
	Unknown ethnicity	27,205	22.28
	Hispanic or Latino	6951	5.69

### Temporal distribution of postoperative complications

**Fig 1:**
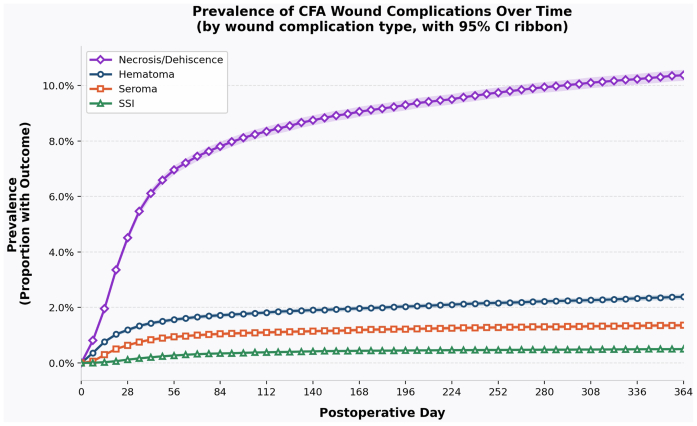
Prevalence of common femoral artery (*CFA*) wound complications over time. Kaplan-Meier-derived cumulative prevalence of each wound complication type after CFA exposure, displayed over 364 postoperative days and sampled at weekly intervals. Prevalence was estimated as *1 − S(t)*, where *S(t)* denotes the Kaplan-Meier survival probability at day *t*. *Shaded ribbons* represent 95% confidence intervals (*CIs*). Necrosis/dehiscence demonstrated the highest cumulative prevalence throughout follow-up, reaching approximately 10% by 1 year, while hematoma, seroma, and surgical site infection (*SSI*) each remained below 2.5%.

The peak incidence rate (per 10,000 patients), day of peak incidence, and final prevalence of hematoma, seroma, SSI, and necrosis/dehiscence within 1 year are summarized in Table III. The cumulative prevalence of each complication over the first year after groin incision is depicted in Fig 1. Although hematoma, seroma, and SSI reached a relative plateau in prevalence by 56 days postoperatively, necrosis/dehiscence continued to increase throughout the entirety of the first year postoperatively. Necrosis/dehiscence had the highest prevalence, with a final prevalence rate of 10.38%.

**Table III:** Prevalence of hematoma, seroma, surgical site infections (*SSIs*), and skin necrosis/wound dehiscence development at 1 year postoperatively

Postoperative complication	Peak incidence rate (per 10,000)	Day of peak incidence	Final prevalence
Hematoma	6.6	12	2.38
Seroma	5.1	13	1.36
SSI	1.1	44	0.50
Skin necrosis/wound dehiscence	23.6	14	10.38
Total sample size = 129,774

### Association with antiplatelet and anticoagulant therapy

Among the 4375 patients who developed hematoma, antiplatelet or anticoagulant therapy (aspirin, clopidogrel, ticagrelor, prasugrel, rivaroxaban, apixaban, or warfarin) within 7 days postoperatively was associated with increased hematoma risk (RR, 1.48; 95% CI, 1.30-1.68; *P* < .0001). Between postoperative days 14 and 21, risk was further elevated (RR, 3.52; 95% CI, 1.76-7.03; *P* = .0002). A separate analysis was conducted for heparin use only (RR, 3.27; 95% CI, 2.52-4.25; *P* < .0001). RRs for various medication combinations are detailed in Table IV.

**Table IV:** Relative risk ratios (*RRs*) of patients who developed hematomas postoperatively and took various specific combinations of antiplatelet/anticoagulant therapies in the 14 days after groin incision

Antiplatelet/anticoagulant combinations	RR in the first week after groin incision	(95% CI) *P* value	RR 14-21 days after groin incision	(95% CI) *P* value
Aspirin alone	1.17^a^	(1.02-1.34) *P* = .03	1.63	(0.82-3.21) *P* = .16
Aspirin and clopidogrel	1.23^a^	(1.07-1.42) *P* = .004	2.02^a^	(1.06-3.88) *P* = .03
Aspirin, clopidogrel, and apixaban	1.32^a^	(1.15-1.51) *P* = .0001	2.71^a^	(1.42-5.17) *P* = .002
Aspirin and apixaban	1.25^a^	(1.09-1.43) *P* = .001	2.11^a^	(1.11-4.02) *P* = .02
Clopidogrel and apixaban	0.92	(0.78-1.08) *P* = .29	2.42^a^	(1.47-4.00) *P* = .0004
Aspirin, clopidogrel, and rivaroxaban	1.28^a^	(1.11-1.47) *P* = .0007	1.64	(0.85-3.16) *P* = .14
Aspirin and rivaroxaban	1.23^a^	(1.07-1.41) *P* = .004	1.37	(0.70-2.67) P = .36
Warfarin alone	0.73^a^	(0.57-0.92) *P* = .007	0.79	(0.44-1.43) *P* = .43
Aspirin and warfarin	1.19^a^	(1.06-1.34) *P* = .005	1.60	(0.85-3.03) *P* = .15
Aspirin, clopidogrel, and warfarin	1.26^a^	(1.11-1.42) *P* = .0003	2.12^a^	(1.13-4.00) *P* = .018
Heparin	3.27	(2.52-4.25) *P* < .0001	Sample size too small for analysis	

*CI*, Confidence interval.

a Statistically significant.

### Long-term outcomes after any postoperative complication

Patients who experienced at least one postoperative complication were compared with those who did not develop any complication. We assessed the 1-year and 5-year incidences of AKI, acute respiratory failure (ARF), cerebrovascular accident (CVA), mortality, limb loss, and ST-elevation myocardial infarction (STEMI). These data are provided in Supplementary Table IV (online only).

The presence of any postoperative complication significantly increased the 1-year incidence of AKI and limb loss, as well as the 5-year incidence of limb loss, ARF, AKI, and CVA, and was associated with a modest but statistically detectable increase in 5-year mortality (limb loss: RR 3.61; 95% CI, 3.37-3.87; *P* < .0001; ARF: RR 1.28; 95% CI, 1.22-1.35; *P* < .0001; AKI: RR 1.28; 95% CI, 1.22-1.34; *P* < .0001; CVA: RR 1.09; 95% CI, 1.01-1.17; *P* = .027; mortality: RR 1.03; 95% CI, 1.00-1.07; *P* = .044).

**Fig 2:**
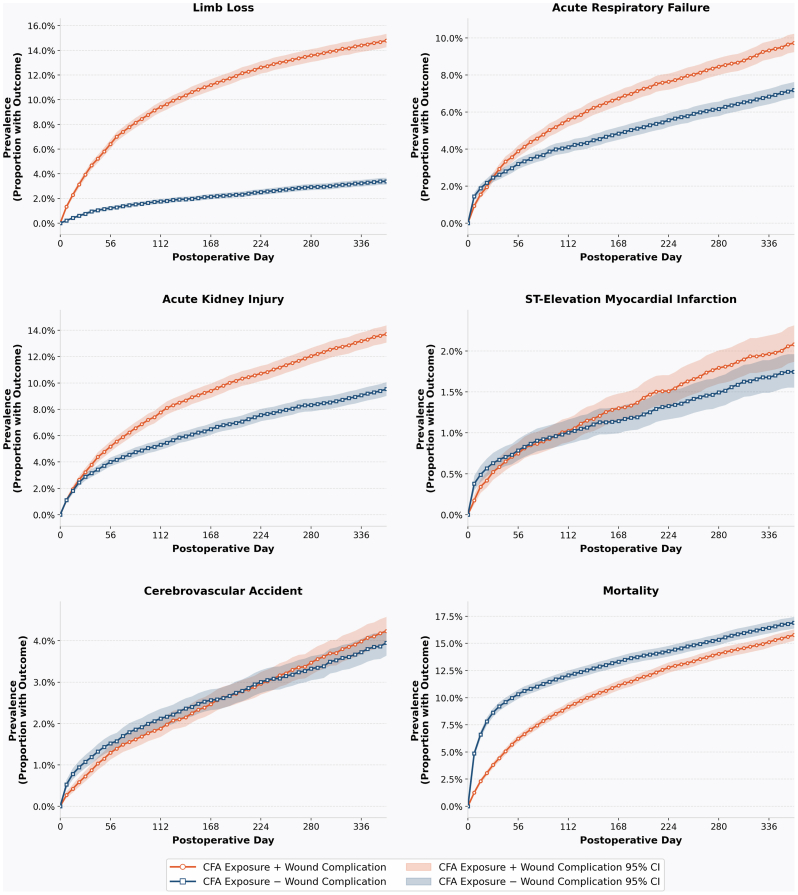
Cumulative prevalence of major sequelae following common femoral artery (*CFA*) exposure, stratified by wound complication status. Kaplan-Meier-derived cumulative prevalence of six postoperative sequelae (limb loss, acute respiratory failure [ARF], acute kidney injury [AKI], ST-elevation myocardial infarction [STEMI], cerebrovascular accident [CVA], and mortality) over 364 postoperative days, compared between patients with CFA exposure and wound complication (*orange*) vs CFA exposure without wound complication (*blue*). *Shaded ribbons* represent 95% confidence intervals (*CIs*). Prevalence was sampled at weekly intervals beginning at day 0. Wound complication was associated with higher cumulative prevalence of limb loss, ARF, AKI, and STEMI, whereas mortality was higher in the CFA exposure without wound complication cohort, consistent with a survival bias in the wound complication group.

Among these outcomes, limb loss posed the greatest long-term risk, followed by AKI and ARF. The 1-year prevalence data of all six long-term adverse outcomes are visualized in Fig 2 to highlight the temporal distribution of these sequelae.

**Fig 3:**
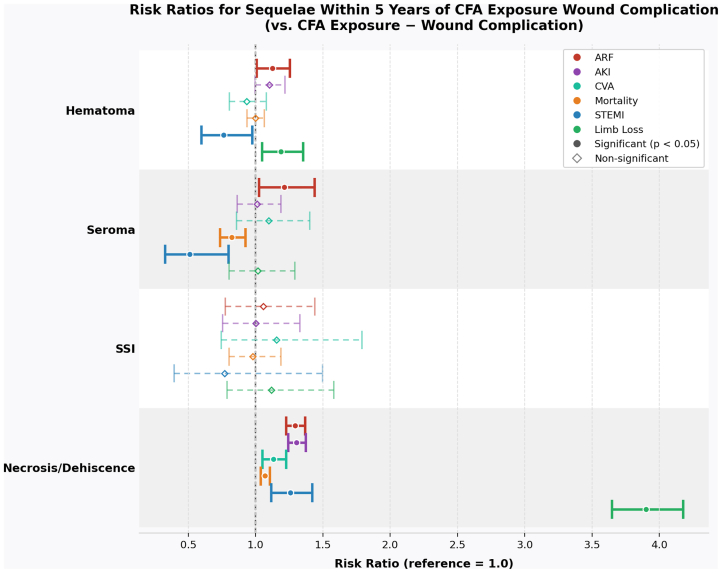
Risk ratios (RRs) for major sequelae within 5 years of common femoral artery (*CFA*) exposure, stratified by wound complication type. Forest plot displaying RRs with 95% confidence intervals (CIs) for six postoperative sequelae (acute renal failure [*ARF*], acute kidney injury [*AKI*], cerebrovascular accident [*CVA*], mortality, ST-elevation myocardial infarction [*STEMI*], and limb loss) within 5 years of CFA exposure, stratified by wound complication type (hematoma, seroma, SSI, and necrosis/dehiscence). The reference group is CFA exposure without wound complication. Each wound complication type is displayed as a labelled row, with the RR for each sequela shown as offset CI bars. *Filled circles* denote statistically significant associations (*P* < .05); *hollow diamonds* denote nonsignificant associations. *Dashed lines* indicate nonsignificant CIs. Necrosis/dehiscence was associated with a markedly elevated risk of limb loss (RR ≈ 3.9); associations for other complication-sequela pairs were more modest in magnitude.

To further delineate the contribution of each complication type, Fig 3 presents the 5-year relative risks and 95% CIs of each medical sequela as a function of individual postoperative complications (hematoma, seroma, SSI, and necrosis/dehiscence). This detailed breakdown highlights which complications are most strongly associated with long-term adverse outcomes. Necrosis/dehiscence emerged as the strongest predictor across all outcomes. Interestingly, hematoma was associated with a significantly decreased risk of STEMI, whereas seroma was associated with significantly decreased risks of both mortality and STEMI.

### Outcomes after surgical correction of postoperative complications

We next compared patients who underwent surgical correction of a postoperative complication within 3 months of its occurrence with those who did not undergo corrective intervention. Relative risks of AKI, ARF, CVA, myocardial infarction, limb loss, and mortality within 6 months of the corrective procedure are summarized in Supplementary Table V (online only).

**Fig 4:**
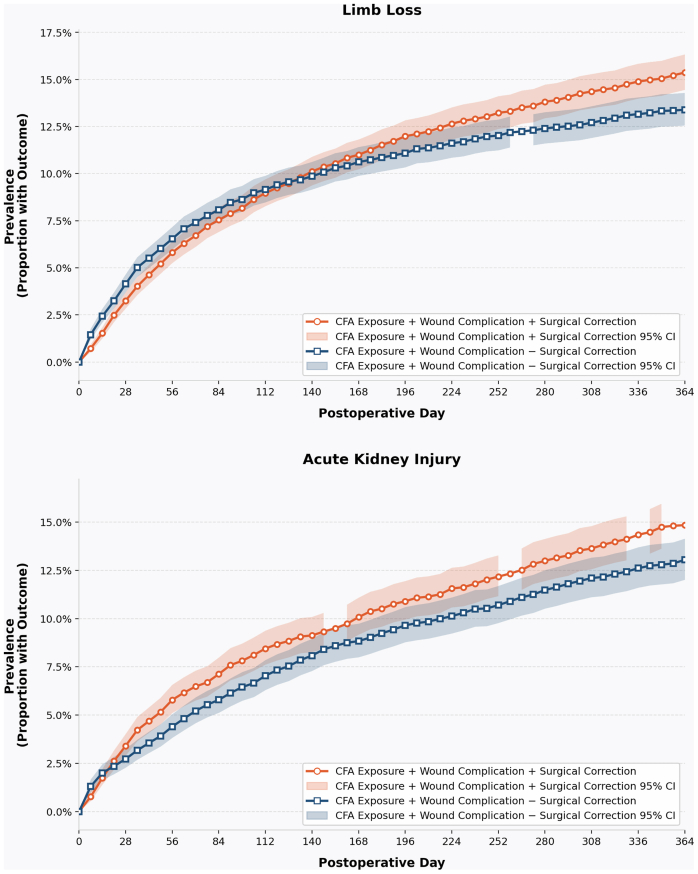
Cumulative prevalence of acute kidney injury (AKI) and limb loss following common femoral artery (*CFA*) wound complication, stratified by surgical revision status. Kaplan-Meier-derived cumulative prevalence of acute AKI (*top*) and limb loss (*bottom*) over 364 postoperative days, compared between patients who underwent surgical correction of their CFA wound complication (*orange*) vs those who did not (*blue*). *Shaded ribbons* represent 95% confidence intervals (*CIs*). Prevalence was sampled at weekly intervals beginning at day 0. Gaps in CI ribbons at select time points reflect TriNetX small-cell privacy suppression, wherein confidence bounds are withheld when the contributing patient count falls below the minimum reporting threshold; point estimates remain displayed at these time points.

Patients requiring corrective surgery had increased 1-year and 5-year risks of limb loss (1-year RR, 1.10; 95% CI, 1.01-1.20; *P* < .0001; 5-year RR, 1.23; 95% CI, 1.15-1.33; *P* < .0001) and AKI (1-year RR, 1.19; 95% CI, 1.06-1.33; *P* = .002; 5-year RR, 1.14; 95% CI, 1.06-1.24; *P* = .0007) compared with patients without wound complications. The 1-year prevalence data of AKI and limb loss in this cohort are displayed in Fig 4. No other outcomes were significantly increased.

## Discussion

### Key findings

Among the four postoperative complications studied (hematoma, seroma, SSI, and wound necrosis/dehiscence), wound necrosis/dehiscence was the most prevalent and exhibited the strongest association with long-term adverse outcomes. The magnitude of risk varied by complication type, postoperative timeframe, and whether a corrective procedure was performed. Secondary procedures after CFA exposure further increased the incidence of high-acuity sequelae. Given the established association between redo groin exploration and wound morbidity, our secondary analysis, restricted to patients undergoing repeat groin operations, offers additional insight into this subgroup. Although exploratory, these findings suggest that the cumulative operative burden may further influence long-term systemic and limb-related outcomes.

### Paradoxical or unexpected findings

Some counterintuitive results were observed. The development of a seroma was associated with a significantly lower risk of 5-year mortality (RR, 0.82; 95% CI, 0.74-0.92; *P* = .0008) and STEMI (RR, 0.51; 95% CI, 0.33-0.80; *P* = .003). Similarly, postoperative hematoma was associated with a lower 5-year risk of STEMI (RR, 0.76; 95% CI, 0.60-0.97; *P* = .03). These paradoxical associations are unlikely to represent a true protective biological effect. Rather, they most plausibly reflect intensified early postoperative surveillance, closer clinical follow-up during the first 30 days, and more aggressive medical optimization in patients who experience early groin complications. Increased provider contact during this vulnerable period may facilitate timely adjustment of antithrombotic therapy, optimization of cardiovascular risk factors, and early detection of systemic complications. Accordingly, these observations should be interpreted as highlighting the potential benefit of structured early follow-up and sustained postoperative medical management, rather than as evidence of reduced intrinsic risk. These associations may also reflect unmeasured factors, selection bias, or coding variability.^4^^,^^6^^,^^12^ Patients without complications are often discharged once clinically appropriate and may be lost to follow-up, whereas hospitalized patients receive ongoing surveillance, such as telemetry for known cardiac disease, which may influence event detection.^6^^,^^12^

### Temporal patterns of long-term adverse outcomes

The majority of adverse outcomes occurred within the first 2 months postoperatively, across all wound complications and long-term sequelae analyzed. This timing likely reflects perioperative monitoring, patient adherence to postoperative recommendations, and the latency of certain vascular or infectious processes.^3^^,^^12^ Incidence decreased exponentially as time after the index surgery continued.

### Risk factors and comorbidities

Patients undergoing vascular procedures are at an increased risk for wound complications and subsequent adverse outcomes due to the comorbid conditions necessitating such procedures, in contrast with healthier individuals undergoing CFA exposure.^4^^,^^5^ Established risk factors for groin wound complications include older age, female sex, preoperative and postoperative antithrombotic therapy, procedural complexity, redo groin exploration, smaller body surface area, and comorbidities such as diabetes, anemia, and chronic obstructive pulmonary disease.^2^^,^^4^ Our study partially controlled for these factors using propensity score matching for age, sex, race, ethnicity, hypertension, diabetes, ischemic heart disease, end-stage renal failure, and tobacco use. Despite this adjustment, postoperative complications remained the strongest predictor of high-acuity sequelae.

### Mechanisms and pathophysiology

Although associations between wound complications and adverse outcomes were observed, the underlying mechanisms remain incompletely understood. For AKI, proposed pathways include embolization of atheromatous debris during repeat vascular interventions, causing renal ischemia and tubular injury.^4^^,^^13^ Hemodynamic instability from hemorrhage or infection may further exacerbate renal injury by reducing perfusion.

Postoperative wound complications may also trigger systemic inflammation and a prothrombotic state, contributing to ARF, CVA, and myocardial infarction.^4^^,^^6^ Infected or dehisced wounds can induce a cytokine response, resulting in endothelial dysfunction and impaired oxygen delivery to vital organs.^5^ Limb loss may result from local ischemia in the setting of graft compromise, infection, or repeated interventions.^9^^,^^14^ Repeated procedures to correct complications can amplify physiological stress, increasing the likelihood of multiorgan dysfunction. Seroma formation may also contribute to local tissue tension, delayed healing, and downstream complications.^15^ These mechanisms highlight how local groin complications can act as both markers and mediators of systemic adverse events in high-risk vascular patients.

### Limitations

As a retrospective analysis of a large, deidentified EHR database, this study is subject to the inherent limitations of code-based outcome ascertainment, residual confounding despite propensity score matching, and limited granularity regarding operative technique and wound severity. Coding errors, variability across institutions, and incomplete capture may result in the misclassification or underestimation of complication rates.^1^^,^^3^ This factor led to an inability to discern certain operative characteristics, such as the orientation of the incision performed. Therefore, we were unable to distinguish which cases were completed through an oblique incision as opposed to a longitudinal incision. Because administrative coding does not reliably capture wound depth or severity, the analytic separation of SSI and necrosis/dehiscence was performed to allow clearer stratification and reduce potential misclassification bias. Additionally, further delineation of postoperative wound complication, such as distinguishing seroma from postoperative lymphatic leak, was not conducted to preserve the specificity of a seroma as a groin wound complication. Second, complications were treated as a binary variable, without accounting for severity, multiplicity, or cumulative effects, potentially underestimating the impact of complex postoperative courses. In the process of propensity score matching, only demographics, comorbidities, and social factors were included to reduce the confounding effect. Given that this study involved a separate analysis of patients who were undergoing reoperation for a groin wound complication, we did not include reoperation as a variable in propensity score matching. Third, wound necrosis and dehiscence were grouped together, which may obscure differences in their individual contributions to outcomes. Finally, residual confounding is possible despite propensity score matching, and clinical details such as operative technique, wound care practices, and outpatient follow-up were unavailable.

Despite these limitations, the large, multicenter nature of this dataset provides a unique opportunity to quantify the long-term impact of groin wound complications and identify high-risk patients.

### Clinical implications and future directions

Our findings emphasize the clinical relevance of postoperative groin wound complications as markers of increased systemic and limb risk after CFA exposure. The temporal patterns observed in this study suggest that the period immediately after discharge represents a critical window during which complications emerge and may influence downstream outcomes. Accordingly, closer postoperative surveillance during the first 2 months after surgery may facilitate the earlier identification and management of hematoma, seroma, SSIs, and wound necrosis/dehiscence.

Although operative and perioperative factors such as incision orientation, hemostasis, and antithrombotic management remain important considerations, these factors were not directly evaluated in this analysis and should be interpreted in the context of prior literature.^14^^,^^16^, ^17^, ^18^ Rather, our results support a framework in which patients with early wound complications, particularly those requiring reintervention, are recognized as a high-risk subgroup who may benefit from more intensive follow-up and coordinated postoperative care. Consistent with prior work, most wound complications occur after hospital discharge, and structured outpatient follow-up during the first 60 days after discharge may improve outcomes.^19^

The findings of this study support a paradigm in which groin wound complications after CFA exposure are treated as early indicators of increased systemic risk. The integration of these observations into risk stratification and postoperative surveillance protocols may enable earlier identification and targeted monitoring of high-risk patients. Future research should focus on the prospective validation of these approaches, the incorporation of risk stratification models, including digital tools such as the Vasculink iPhone application, and the development of EHR-based early warning systems to improve the detection of groin wound complications.^20^

Collectively, these findings highlight specific predictors of long-term adverse outcomes and provide actionable insights for vascular surgeons and health care teams to improve both immediate and downstream patient outcomes.

## Conclusions

In this large, multicenter, retrospective study, wound necrosis/dehiscence was the most prevalent complication after CFA exposure and demonstrated the strongest association with long-term adverse outcomes. Hematoma, seroma, SSIs, and wound necrosis/dehiscence also contributed to downstream risks, including AKI, ARF, CVA, STEMI, limb loss, and mortality, with risk magnitude influenced by complication type, timing, and the need for corrective procedures. Paradoxical associations observed for hematoma and seroma likely reflect heightened monitoring and unmeasured factors, highlighting the complexity of postoperative surveillance. These findings emphasize that postoperative wound complications are not merely local events, but also important predictors of systemic outcomes. The recognition of groin wound complications as predictors of long-term systemic and limb morbidity supports the use of risk-stratified surveillance and intensified early postoperative follow-up to improve patient outcomes. The early identification of high-risk patients, coupled with coordinated outpatient monitoring and timely management of complications, may help to mitigate downstream adverse events in vascular surgery patients.

## Declaration of generative AI and AI-assisted technologies in the writing process

During the preparation of this work the authors used OpenAI's ChatGPT 4.1 and Anthropic's Claude Sonnet 4.6 as adjuncts to enhance readability and clarity. After using these tools/services, the authors reviewed and edited the content as needed and take full responsibility for the content of the publication.

## Author Contributions

Conception and design: KA, DS, MR

Analysis and interpretation: KA, RB, MO, AS, MR

Data collection: KA, MR

Writing the article: KA, RB, MO, DS, MR

Critical revision of the article: KA, RB, MO, AS, MR

Final approval of the article: KA, RB, MO, DS, AS, MR

Statistical analysis: Not applicable

Obtained funding: Not applicable

Overall responsibility: MR

## Funding

None.

## Disclosures

None.
