# Supplementary material for: Postoperative groin wound complications after common femoral artery exposure are associated with increased long-term systemic and limb adverse outcomes in a national propensity-matched analysis
Source: J Vasc Surg Cases Innov Tech. 2026 Apr 30;12(4):102285. doi: 10.1016/j.jvscit.2026.102285 (PMC13234736; doi:10.1016/j.jvscit.2026.102285)
Supplement: Supplementary Tables [file mmc1.docx]

**Supplemental Table I.** ICD-10/CPT/SNOMED codes identifying patients who underwent common femoral artery exposure.

| **Procedure Code** | **Description** |
| --- | --- |
| 34812 | Open femoral artery exposure for delivery of endovascular prosthesis, by groin incision, unilateral |
| 405463009 | Femoral artery bypass |
| 34714 | Open femoral artery exposure with creation of conduit for delivery of endovascular prosthesis or for establishment of cardiopulmonary bypass, by groin incision, unilateral |
| 35539 | Bypass graft, with vein; aortofemoral |
| 35521 | Bypass graft, with vein; axillary-femoral |
| 35533 | Bypass graft, with vein; axillary-femoral-femoral |
| 34832 | Open repair of infrarenal aortic aneurysm or dissection, plus repair of associated arterial trauma, following unsuccessful endovascular repair; aorto-bifemoral prosthesis |
| 35141 | Direct repair of aneurysm, pseudoaneurysm, or excision (partial or total) and graft insertion, with or without patch graft; for aneurysm, pseudoaneurysm, and associated occlusive disease, common femoral artery (profunda femoris, superficial femoral) |
| 35142 | Direct repair of aneurysm, pseudoaneurysm, or excision (partial or total) and graft insertion, with or without patch graft; for ruptured aneurysm, common femoral artery (profunda femoris, superficial femoral) |
| 35363 | Thromboendarterectomy, including patch graft, if performed; combined aortoiliofemoral |
| 35371 | Thromboendarterectomy, including patch graft, if performed; common femoral |
| 35372 | Thromboendarterectomy, including patch graft, if performed; deep (profunda) femoral |
| 35721 | Exploration (not followed by surgical repair), with or without lysis of artery; femoral artery (deprecated 2021) |
| 34201 | Embolectomy or thrombectomy, with or without catheter, femoropopliteal, aortoiliac artery, by leg incision |
| 35540 | Bypass graft, with vein; aortobifemoral |
| 35556 | Bypass graft, with vein; femoral-popliteal |
| 35558 | Bypass graft, with vein; femoral-femoral |
| 35565 | Bypass graft, with vein; iliofemoral |
| 35566 | Bypass graft, with vein; femoral-anterior tibial, posterior tibial, peroneal artery or other distal vessels |
| 35583 | In-situ vein bypass; femoral-popliteal |
| 35585 | In-situ vein bypass; femoral-anterior tibial, posterior tibial, or peroneal artery |
| 35621 | Bypass graft, with other than vein; axillary-femoral |
| 35646 | Bypass graft, with other than vein; aortobifemoral |
| 35647 | Bypass graft, with other than vein; aortofemoral |
| 35654 | Bypass graft, with other than vein; axillary-femoral-femoral |
| 35656 | Bypass graft, with other than vein; femoral-popliteal |
| 35661 | Bypass graft, with other than vein; femoral-femoral |
| 35665 | Bypass graft, with other than vein; iliofemoral |
| 35666 | Bypass graft, with other than vein; femoral-anterior tibial, posterior tibial, or peroneal artery |
| 175689005 | Endarterectomy of femoral artery and patch repair of femoral artery |
| 8409001 | Embolectomy of femoral artery |
| 112828007 | Femoral-popliteal bypass graft |
| 405462004 | Femoral-distal popliteal artery bypass |
| 405463009 | Femoral artery bypass |
| 16589005 | Femoral endarterectomy |
| 35355 | Thromboendarterectomy, including patch graft, if performed; iliofemoral |

**Supplemental Table II.** ICD-10/CPT/SNOMED codes identifying patients who experienced a post-operative complication after common femoral artery exposure.

| **Postoperative Complication Code** | **Description** |
| --- | --- |
| I97.622 | Postprocedural seroma of a circulatory system organ or structure following other procedure |
| L76.34 | Postprocedural seroma of skin and subcutaneous tissue following other procedure |
| M96.843 | Postprocedural seroma of musculoskeletal structure following other procedure |
| L76.32 | Postprocedural hematoma of skin and subcutaneous tissue following other procedure |
| M96.841 | Postprocedural hematoma of musculoskeletal tissue following other procedure |
| I97.63 | Postprocedural hematoma of a circulatory system organ or structure following other procedure |
| T81.42XS | Infection following a procedure, deep incisional surgical site, sequela |
| T81.41XD | Infection following a procedure, superficial incisional surgical site, subsequent encounter |
| T81.41XS | Infection following a procedure, superficial incisional surgical site, sequela |
| T81.42XD | Infection following a procedure, deep incisional surgical site, subsequent encounter |
| T81.40XD | Infection following a procedure, unspecified, subsequent encounter |
| T81.40XS | Infection following a procedure, unspecified, sequela |
| L76.82 | Other postprocedural complications of skin and subcutaneous tissue |
| T81.31XA | Disruption of external operation (surgical) wound, not elsewhere classified, initial encounter |
| I96 | Gangrene, not elsewhere classified |

**Supplemental Table III.** ICD-10/CPT/SNOMED codes identifying the specific operative corrections of post-operative complication after common femoral artery exposure.

| **Operative Correction of Postoperative Complication Code** | **Description** |
| --- | --- |
| 19780006 | Debridement of infection of skin |
| 0JBM0ZZ | Excision of Left Upper Leg Subcutaneous Tissue and Fascia, Open Approach |
| 0JBL0ZZ | Excision of Right Upper Leg Subcutaneous Tissue and Fascia, Open Approach |
| 473218004 | Excisional debridement of wound for infection |
| 225148005 | Surgical debridement of wound |
| 0KTR0ZZ | Resection of Left Upper Leg Muscle, Open Approach |
| 0KTQ0ZZ | Resection of Right Upper Leg Muscle, Open Approach |
| 0KBR3ZZ | Excision of Left Upper Leg Muscle, Percutaneous Approach |
| 0KBQ3ZZ | Excision of Right Upper Leg Muscle, Percutaneous Approach |
| 0KBQ0ZZ | Excision of Right Upper Leg Muscle, Open Approach |
| 0KBR0ZZ | Excision of Left Upper Leg Muscle, Open Approach |
| 0HBHXZZ | Excision of Right Upper Leg Skin, External Approach |
| 0HBJXZZ | Excision of Left Upper Leg Skin, External Approach |
| 118441006 | Evacuation of hematoma |
| 450671005 | Debridement of soft tissue |
| 85875009 | Debridement of wound of skin |
| 97598 | Debridement (eg, high pressure waterjet with/without suction, sharp selective debridement with scissors, scalpel, or forceps), open wound (eg, fibrin, devitalized epidermis and/or dermis, exudate, debris, biofilm), including topical application(s), wound assessment, use of a whirlpool, when performed and instruction(s) for ongoing care, per session, total wound(s) surface area; each additional 20 sq cm, or part thereof (List separately in addition to code for primary procedure) |
| 1020524 | Debridement, muscle and/or fascia (includes epidermis, dermis, and subcutaneous tissue, if performed) |
| 0JDM0ZZ | Extraction of Left Upper Leg Subcutaneous Tissue and Fascia, Open Approach |
| 0JDL0ZZ | Extraction of Right Upper Leg Subcutaneous Tissue and Fascia, Open Approach |
| 0HDJXZZ | Extraction of Left Upper Leg Skin, External Approach |
| 0HDHXZZ | Extraction of Right Upper Leg Skin, External Approach |
| 0HDAXZZ | Extraction of Inguinal Skin, External Approach |
| 11043 | Debridement, muscle and/or fascia (includes epidermis, dermis, and subcutaneous tissue, if performed); first 20 sq cm or less |
| 11042 | Debridement, subcutaneous tissue (includes epidermis and dermis, if performed); first 20 sq cm or less |
| 27603 | Incision and drainage, leg or ankle; deep abscess or hematoma |
| 10140 | Incision and drainage of hematoma, seroma or fluid collection |

**Supplemental Table IV.** Relative risk ratio of all outcomes within 1 year and 5 years of groin incision in patients with postoperative complication. * = statistical significance

| **Outcome** | **Risk Ratio in 1 year** | **95% Confidence Interval, p-value** | **Risk Ratio in 5 years** | **95% Confidence Interval, p-value** |
| --- | --- | --- | --- | --- |
| Limb Loss | 4.43* | (4.41, 4.84)  p < 0.0001 | 3.61* | (3.37, 3.87)  p < 0.0001 |
| Acute Respiratory Failure | 1.42* | (1.31, 1.15)  p < 0.0001 | 1.28* | (1.22, 1.35)  p < 0.0001 |
| Acute Kidney Injury | 1.49* | (1.38, 1.60)  p < 0.0001 | 1.28* | (1.22, 1.34)  p < 0.0001 |
| Cerebrovascular Accident | 1.08 | (0.96, 1.21)  p = 0.19 | 1.09* | (1.01, 1.17)  p = 0.03 |
| Mortality | 0.98* | (0.90, 0.98) | 1.03* | (1.001, 1.07)  p = 0.04 |
| STEMI | 1.23* | (1.05, 1.45)  p = 0.013 | 1.07 | (0.96, 1.20)  p = 0.25 |

**Supplemental Table V.** Relative risk ratio of all outcomes within 1 year and 5 years of operative correction of postoperative complication. * = statistical significance

| **Outcome** | **Risk Ratio in 1 years** | **95% CI** | **Risk Ratio in 5 years** | **95% CI** |
| --- | --- | --- | --- | --- |
| Limb Loss | 1.10* | (1.01, 1.20)  p = 0.03 | 1.23* | (1.15, 1.33)  p < 0.0001 |
| Acute Kidney Injury | 1.19* | (1.06, 1.33)  p = 0.002 | 1.14* | (1.06, 1.24)  p = 0.0007 |
| Acute Respiratory Failure | 1.02 | (0.90, 1.15)  p = 0.79 | 1.04 | (0.95, 1.12)  p = 0.41 |
| STEMI | 1.07 | (0.83, 1.37)  p = 0.61 | 1.10 | (0.92, 1.33)  p = 0.29 |
| Cerebrovascular Accident | 0.99 | (0.83, 1.18)  p = 0.90 | 1.02 | (0.91, 1.15)  p = 0.75 |
| Mortality | 0.97 | (0.90, 1.04)  p = 0.40 | 1.00 | (0.95, 1.04)  p = 0.80 |
